# Study on the influence of slit structure on safe and efficient directional fracture blasting effect

**DOI:** 10.1038/s41598-023-43814-3

**Published:** 2023-10-10

**Authors:** Yingxiang Tian, Yiping Zhang, Huaying Lin, Enan Chi

**Affiliations:** 1 Department of Public Security, Guizhou Police College, Guiyang, 550005 Guizhou China; 2 College of Mining Engineering, Guizhou University, Guiyang, 550025 Guizhou China; 3 Poly Jiulian Holding Group Co., Ltd, Guiyang, 550002 Guizhou China

**Keywords:** Engineering, Civil engineering, Energy infrastructure

## Abstract

In order to study the influence of slit structure on the blasting effect of slit charge, the super dynamic strain test system and photographic equipment were used to study the dynamic response of slit charge blasting and the distribution of blasting cracks under different slit shapes and slit angles. The results show that changing the shape of the slit does not make the energy-gathering and damage-reducing blasting effect of the slit cartridge disappear, but it will affect its effect. Compared with the round hole slit, the peak strain in the slit direction of the strip slit cartridge blast is larger, the directional crack forming is more obvious, and the blasting effect is better. The energy accumulation and loss reduction effect of the blasting of the strip slotted cartridge increase first and then decrease with the increase of the slit angle. When the slit angle is 20°, the energy accumulation and loss reduction effect of strip slit charge blasting are the largest, the distribution of blasting cracks is the best, and the directional fracture blasting effect of slit charge is the best. Finally, based on the research results of model test, combined with the actual situation of the construction site, the slotted cartridge is applied to the pre-splitting blasting of open-pit slope. Compared with the ordinary cartridge pre-splitting blasting, the slope roughness after blasting is reduced by 46.2%, the half-hole rate of blast hole is increased by 20.5%, the blasting vibration intensity is reduced by 60.3%, and the directional fracture control blasting effect is good.

## Introduction

With the wide application of blasting technology in basic projects such as energy mining, tunnels and slopes, this technology has now entered a stage of high-quality development. How to achieve safe and efficient precision blasting technology is the focus of current research in the field of blasting. As one of the high-quality controlled blasting technologies that are mainly promoted and applied, the directional fracture blasting of slotted cartridge is often applied to the controlled blasting of medium-hard rock mass with relatively developed joints and cracks^1^, and its development has attracted much attention. The technology was first proposed by Fourney W.L.and others in the 1970s^2,3^, and was introduced into China in the 1980s. It mainly controls the explosive stress distribution in the blast hole, the gas wedge effect and the quasi-static effect of the explosive gas during the explosive explosion through the slitting device, so that the blasted rock mass can achieve the effect of directional fracture. So far, relevant researchers have carried out a series of studies on its mechanism, engineering application and main influencing factors of blasting effect^4^.

Zhang Zhicheng^5^, Luo Yong^6^, Dai Jun^7,8^ et al. conducted theoretical research on the directional fracture blasting mechanism, crack initiation and crack arrest conditions of slit charge rock, analyzed the mechanical characteristics of initial cracks and crack propagation of slit charge blasting, and found that there is a direct correlation between slit width and directional crack propagation. After that, some scholars studied the blasting mechanism and influencing factors of slotted cartridges by means of experiments. For example, Yang Renshu, Song Junsheng, Wang, Kang and so on comprehensively used high-speed camera, dynamic caustics instrument and high-speed schlieren test system to carry out indoor blasting test, and analyzed the trajectory of shock wave, detonation gas and crack propagation during slit charge blasting. It is found that the explosion shock wave and detonation gas are always preferentially transmitted from the slit direction. The explosion shock wave is the main factor causing the initial crack. The detonation gas plays an important role in the extension of the later crack. The tensile failure of the crack mostly occurs in the near area of the explosion, and the shear failure generally occurs in the middle and far area. At the same time, it also analyzes how the explosion stress wave and the detonation gas promote the expansion and extension of the crack, and deeply studies the directional fracture blasting mechanism of the slit cartridge^9–15^. Jin et al.^16^ accurately described the distribution of blasting cracks in slit charge by means of CT scanning. Yue Zhongwen, Yang Renshu et al.^17,18^ studied the effect of radial uncoupling coefficient of charge on the blasting effect of slit charge through model tests and found that uncoupled charge produced fewer secondary cracks than coupled charge blasting, which was more conducive to protecting the integrity of rock mass in non-slit direction. When the uncoupling coefficient was 1.67, the directional fracture blasting effect of slit charge was good. Yang Guoliang et al.^19^ studied the influence of axial decoupling coefficient of charge on the blasting effect of slit charge by means of dynamic strain test system and high-speed camera. It was found that when the axial decoupling coefficient was 1.5, the strain peak ratio of slit direction to vertical slit direction was the largest, and the blasting effect was the best. Shan Xingliang et al.^20^ obtained the best non-coupling coefficient and slit width of the slit cartridge through the concrete model blasting experiment. Based on the model test results, the blasting test was carried out in the soft rock roadway of Dayan mining area, and the half eye mark rate was more than 83%, and the overbreak and underbreak was less than 100 mm. In addition, due to the limitations of the experiment, some scholars have carried out numerical simulation and application research on the directional fracture blasting of slotted cartridge. For example, Ding et al.^21^ studied the influence of multiple slits on the stress evolution and blasting vibration of slotted cartridge blasting through numerical simulation. Shen Tao^22^ constructed a slotted cartridge blasting model by means of numerical simulation, and analyzed the expansion process of detonation products in the initial stage of explosion and the damage process of blast hole wall rock caused by explosion load in the middle stage of explosion. Zhang Shengli^23^ selected the best slit width of PVC pipe slit cartridge by means of ANSYS software simulation, and applied the research results to the field blasting of roadway. The results show that the use of slit cartridge can increase the hole spacing and obtain good pre-splitting blasting effect. Yang and Guo applied the directional fracture blasting technology of slotted cartridge to roadway blasting. The directional fracture effect is good, which can effectively reduce the damage of blasting to the surrounding rock of roadway and reach the pressure relief effect^24,25^.

**Figure 1:**
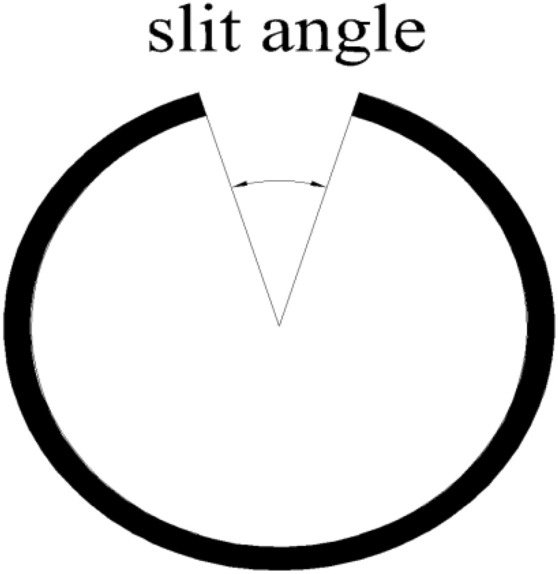
Slit angle diagram.

In summary, compared with ordinary cartridge blasting, the directional fracture effect of rock after slotted cartridge blasting is better and can better reduce the damage of protected rock mass. At present, the research on slotted cartridge blasting is mostly biased towards its energy accumulation mechanism and the mechanical analysis of the start and end of blasting rock cracks, which has laid a certain theoretical foundation in this regard^26^; however, there are few studies on the important factors affecting the blasting effect, such as the decoupling coefficient of charge and the slit structure, and there are even fewer studies on the slit angle and slit shape. In addition, the author also found that when using common similar model tests and numerical simulation methods to study the influence of slit width on the blasting effect of slit charge, it is difficult to accurately apply the optimal slit width value obtained in the study to actual engineering blasting, which is not conducive to the practical application and industrialization of research results.Therefore, it is proposed to use the central angle corresponding to the slit (hereinafter referred to as the ‘slit angle’ as shown in Fig. 1) instead of the ‘slit width’ for corresponding research, so that the research results can be more accurately and effectively applied to engineering blasting.

In this paper, theoretical analysis and model test are used to reveal the influence of slit shape and slit angle on blasting stress and strain, blasting crack, directional energy accumulation and loss reduction of slit cartridge.Then according to the research results, the best slit structure is determined and applied in the field.

## Theoretical analysis of rock breaking by slotted cartridge blasting

### Principle of blasting action of slotted cartridge

**Figure 2:**
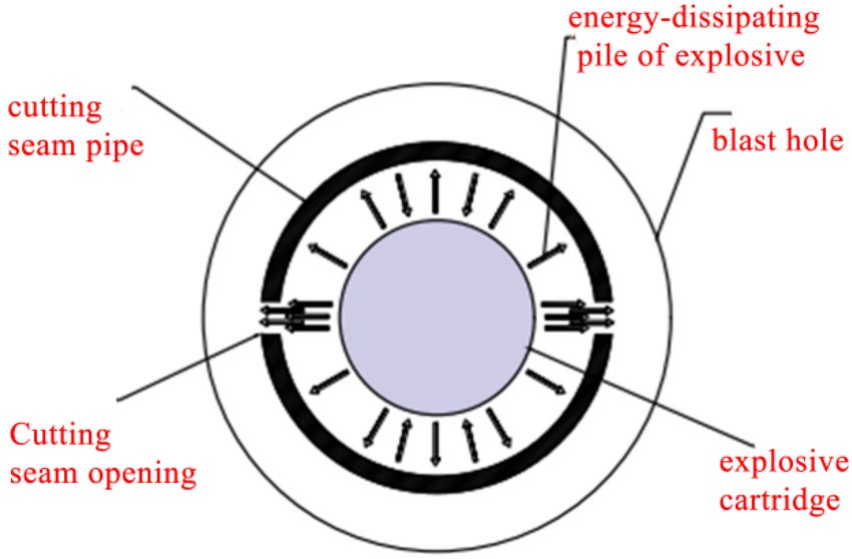
Slit cartridge blasting principle diagram.

Slotted cartridge blasting refers to the purposeful controlled blasting construction technology by loading ordinary explosives into slotted tubes with certain tensile and compressive strength and a certain number of slits^27,28^. The principle of action is shown in Fig. 2. When the explosive in the slotted cartridge is detonated, the generated detonation gas and explosion shock wave will be transmitted around the explosive as the center. In the slit direction, the diversion effect of the slit will make most of the blasting energy preferentially transmitted in this direction, enhance the rock breaking ability in this direction, and accelerate the formation of initial directional cracks. In addition, due to the wrapping effect of the slit tube on the detonation gas and the reflection effect of the shock wave in the non-slit direction, some of the blasting energy originally transmitted in the non-slit direction is transferred to the slit direction to promote the initial crack to further expand and extend. In the non-slitting direction, the transmission of blasting shock wave will be reflected and refracted when it encounters the slit tube, and the transmission of blasting gas will also be constrained by the slit tube, resulting in the blasting energy in this direction acting on the hole wall after the double weakening of the slit tube and air, which reduces the damage of blasting energy to rock mass. So as to achieve the effect of directional fracture blasting. According to the principle of blasting, it can be seen that under the condition of fixed explosive charge, the slit structure is an important factor affecting the directional fracture blasting effect of slit charge.

### Analysis of rock breaking mechanism by directional fracture of slotted cartridge blasting

#### Transmission of blasting stress wave of slotted cartridge

Combined with the existing research, it can be seen that^29,30^, uncoupled charge is more conducive to controlling the distribution and transmission of blasting energy. When the slotted cartridge charge is uncoupled charge, the hole wall pressure value is:

1 $$P = \frac{Pe}{2}\left( {\frac{{r_{a} }}{{r_{b} }}} \right)^{\alpha } n$$

where *Pe* is the detonation pressure of the explosive, MPa; *r*_*a*_ is the radius of the drug package, m ;*r*_*b*_ is the hole radius, m ; *n* is the pressure increase multiple when the detonation product impacts the hole wall, generally 8–11, *α* is the undetermined coefficient, which is related to the shape of the charge.

According to the stress wave theory, the peak pressure of the wave is used as a reference for wave attenuation. The relationship between the attenuation of the stress wave and the distance is^29^:

2 $$\sigma_{r} = \frac{P}{{l^{\omega } }}Z$$

In the formula: *σ*_*r*_ is the stress peak value of any point in the rock, MPa; *Z* is the energy-gathering coefficient, *l* = r/r_b_, *r* is the axial distance from any point of the rock mass to the center of the blast hole, m. *ω* is the attenuation coefficient of stress wave.

#### Crack formation and expansion

According to the theory of rock fracture mechanics, it can be seen that for the brittle material of rock, when the slotted cartridge blasting is used, there are two main rock breaking methods to promote the formation of initial cracks in the hole wall rock in the slit direction^31–33^. First, due to the special structure of the slotted pipe, the size and time of the blasting shock wave acting on the hole wall in the slit direction and the non-slit direction are different, and the pressure difference is formed at the hole wall in the slit direction; Under the action of shear stress, the hole wall rock shear failure occurs. secondly, when the blasting energy transmitted preferentially from the slit direction acts on the hole wall, the circumferential tensile stress generated will destroy the rock. Therefore, it can be concluded that at least one of the following two conditions should be satisfied to form the initial crack^6^.

3 $$P > S_{dt} \left( {1 - \mu } \right)/\mu$$

4 $$P > \left( {C - \tau } \right)\left( {1 - \mu } \right)/\mu \tan \varphi$$

In the formula: *P* is rock pore wall pressure, MPa; *u* is the Poisson's ratio of rock; *C* is rock cohesion, MPa; *φ* is the rock friction angle, °; *S*_*dt*_ is the dynamic uniaxial tensile strength of rock, MPa ; *τ* is the shear stress at the hole wall in the slit direction, MPa.

When the initial crack is formed, whether the crack can continue to expand under the quasi-static action of stress wave and explosive gas depends on whether the stress intensity factor *K*_*I*_ at the crack tip is greater than the fracture toughness *K*_*IC*_ of rock. When *K*_*I*_ ≥ *K*_*IC*_, the crack continues to extend, otherwise it stops extending^34–36^. The calculation formula of *K*_*I*_ is^8^:

5 $$K_{I} = PF\sqrt {\pi \left( {r_{b} + a} \right)}$$

where *F* is the correction factor of stress intensity factor, *F* = [(*r*_*b*_ + *a*)/*r*_*b*_]; *a* is the crack propagation length, m. The initiation condition is substituted into formula (5) to obtain the critical force *P*, formula (6) to maintain the continuous expansion of cracks. If the rock pore wall pressure is less than this critical value, the crack stops expanding.

6 $$P \ge \frac{{K_{IC} }}{{F\sqrt {\pi \left( {r_{b} + a} \right)} }}$$

In the formula, *K*_*IC*_ = 0.141*σ*_*c*_^1.15^, MPa·m^0.5^ is obtained by a large number of tests and analysis of Yangtze River Hydropower Research Institute. Where *σ*_*c*_ is the uniaxial compressive strength of rock, MPa.

According to the blasting mechanism and formula (1)–(6) of slotted cartridge, it can be seen that the peak pressure of hole wall and the propagation of stress wave in the direction of slit are closely related to the structure of slotted cartridge. It directly affects the formation and expansion of directional cracks in rock, so it is of great significance to study the influence of slit shape and slit angle on the blasting effect of slit cartridge.

## Slotted cartridge blasting test

In order to study the influence of slit shape and slit angle on blasting effect, the control variable method was used to analyze the explosion stress and strain distribution and rock crack propagation in slit direction and non-slit direction. The experiment was carried out in two steps. Firstly, the blasting test of slotted cartridge with different slotted shapes was carried out. According to the test results, the slotted shape with the best blasting effect was selected, and then the influence of slotted angle on the directional fracture blasting effect of slotted cartridge was further studied. The test process is as follows.

### Model test block and slit charge making

#### Concrete model making

**Figure 3:**
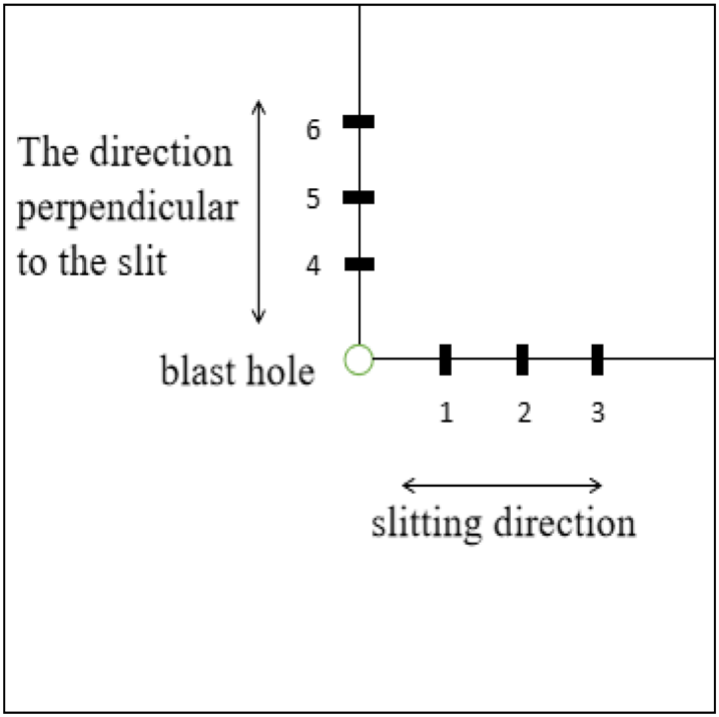
Strain gauge measuring point layout diagram.

**Figure 4:**
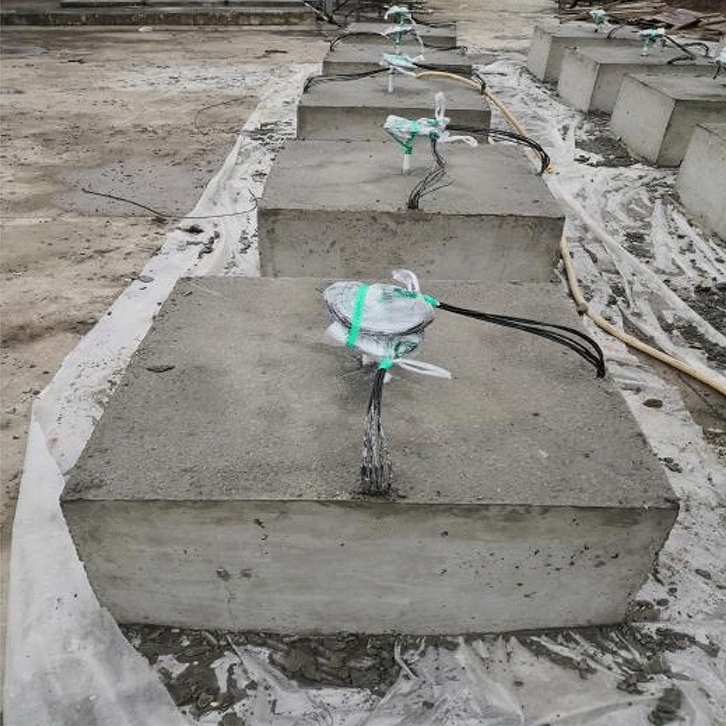
Concrete blasting model test block.

Because the slotted cartridge controlled blasting technology is mostly used in medium-hard rock blasting, the rock compressive strength of this kind of rock mass is generally equal to or greater than 60 MPa. In order to ensure the validity of the test results, the model test block is poured with C60 concrete, and its length, width and height are 1 m × 1 m × 0.5 m. A circular hole with a diameter of 25 mm and a depth of 250 mm is reserved in the center of the test block. When pouring the concrete model specimen, the strain brick is embedded in the fixed position of the concrete model specimen. The specific measuring point arrangement is shown in Fig. 3, and 6 measuring points are arranged for each concrete model specimen. Two strain gauges with the same arrangement are pasted on each measuring point to reduce the error and ensure the reliability of the measured data. The connection mode of the strain gauge is 1/4 bridge. The finished model test block is shown in Fig. 4. After the uniaxial compression test of the standard specimen, the compressive strength of the specimen is 68 MPa, which meets the requirements of the test design.

#### Production of slit cartridges with different slit structures

**Figure 5:**
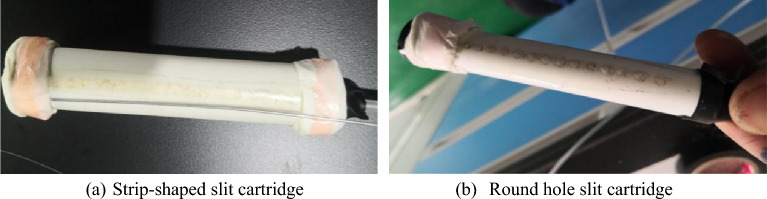
Physical diagram of cutting seam medicine package.

The slit pipe is made of PVC pipe. The outer diameter of the slit pipe is 16 mm, the inner diameter is 14 mm, and the length is 12 cm. Firstly, Strip-shaped slit tubes and round-hole slit tubes with a slit angle of 20° were fabricated. After the first step of the test, the best slit shape was selected. On this basis, four kinds of slit tubes with slit angles of 10°, 20°, 30°and 40° were made respectively. In order to facilitate the charge, the ammonium nitrate expanded explosive was used in the test. The charge amount of each slit tube was 10 g, and the total length of the charge was 10 cm. Some finished slit cartridges are shown in Fig. 5, and the slit cartridges were detonated by detonator with shock-conducting tube.

### Test system and test process

**Figure 6:**
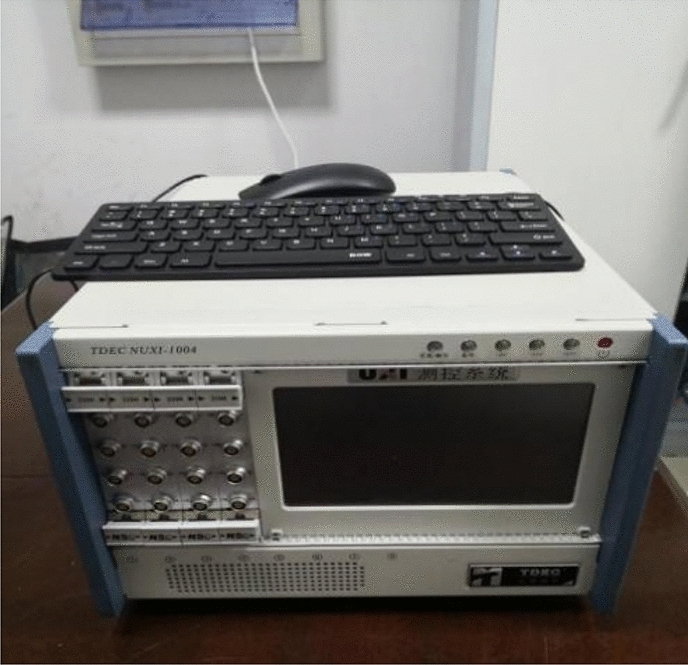
Super dynamic strain gauge test system.

The test system is composed of NUXI-1004 super dynamic strain tester and photographic equipment. The super dynamic strain tester is used for signal conditioning and data acquisition. The equipment is shown in Fig. 6.

**Figure 7:**
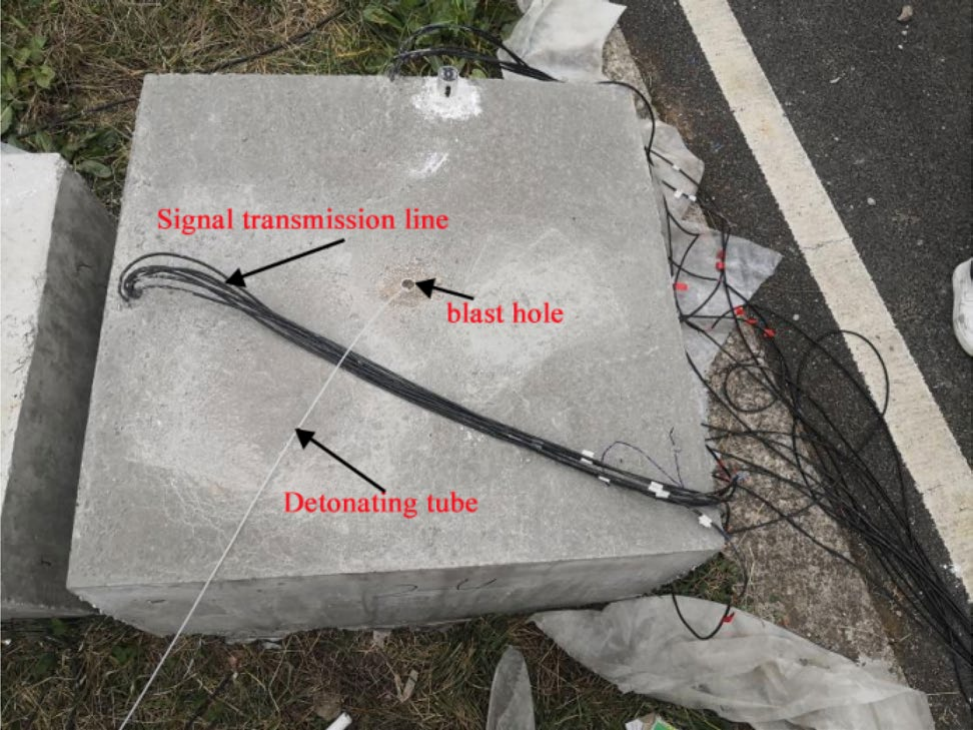
Charge model test block before blasting.

**Figure 8:**
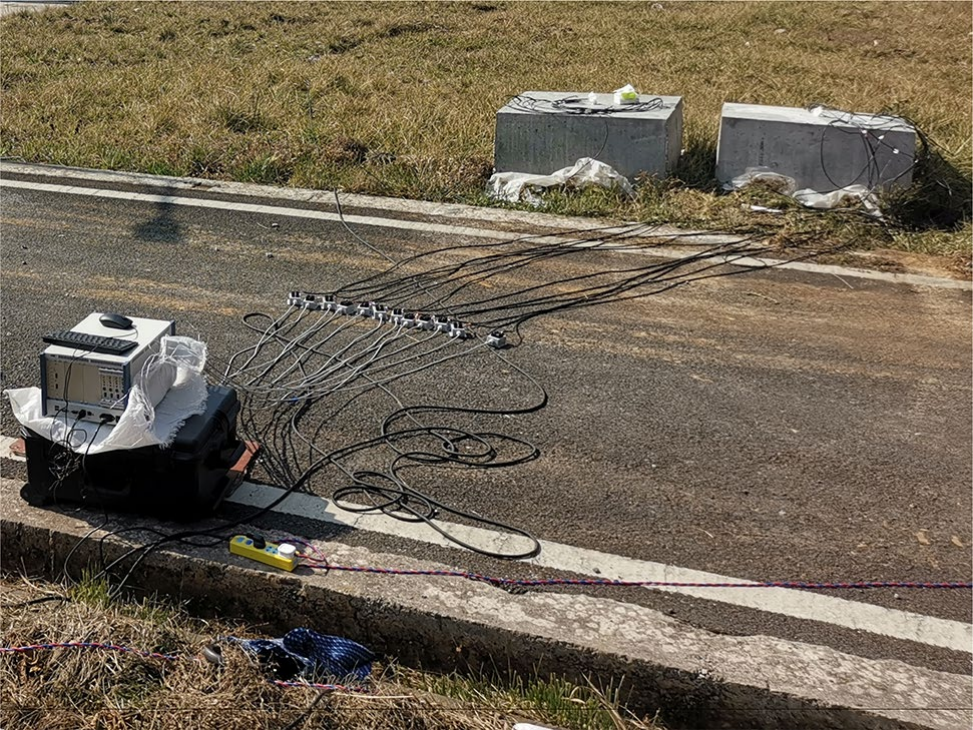
Super dynamic strain test system test diagram.

Before the test, the circular hole-shaped and strip-shaped slotted cartridge blasting model test blocks were marked as No.1 test block and No. 2 test block respectively, and the remaining model test blocks were numbered from small to large according to the slit angle. Then, the uncoupled center charge of the slotted cartridge is carried out. After the charge is completed, the blast hole is blocked by fine sand, and the model specimen to be blasted is obtained as shown in Fig. 7. After that, the super dynamic strain test system was opened, and the blasting was carried out in turn according to the model serial number. The strain value generated during blasting was saved in time after blasting, and the distribution of blasting cracks in each model test block was photographed and recorded. The data acquisition process of the super dynamic strain test system is shown in Fig. 8.

## Slit charge blasting test results and analysis

The stress–strain value and crack distribution of rock mass blasting are taken as the internal and external manifestations of blasting energy rock breaking respectively, and they are analyzed to evaluate the directional fracture blasting effect of slit charge in each model test. In addition, according to the relationship between stress and strain, it can be seen that the variation law of blasting stress and strain in the experiment is consistent, so the blasting strain is taken as the representative to analyze the variation law when analyzing the blasting stress and strain.

### Analysis of the influence of slit shape on blasting effect

#### Dynamic strain analysis of slit charge blasting with different slit shapes

**Figure 9:**
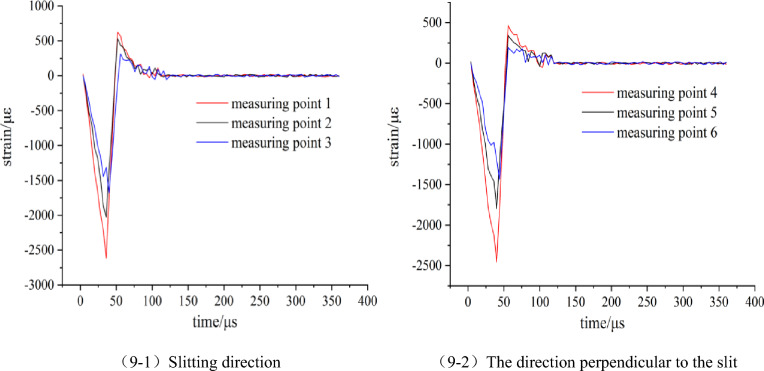
Strain waveform diagram of model 1 (Round hole shaped slit).

**Figure 10:**
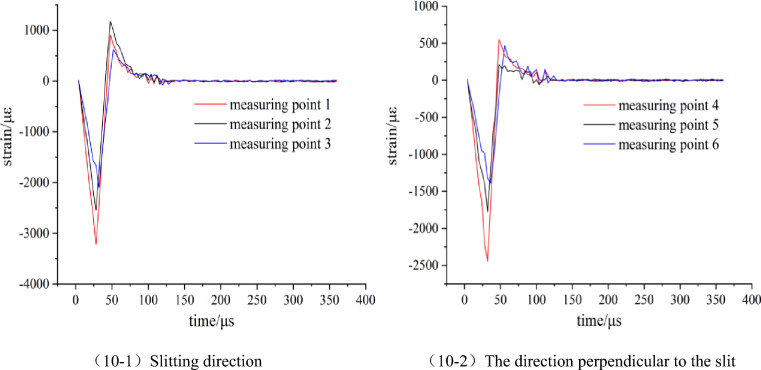
Strain waveform diagram of model 2 (Strip shape slit).

The explosion strain data generated by the blasting of No.1 and No.2 model tests were collected by the ultra-dynamic strain test system. The data were processed and the strain time history curve was obtained as shown in Figs. 9 and 10.

According to Figs. 9 and 10, the strain peaks in the slit direction of the two model tests are larger than those in the vertical slit direction, and the arrival time of the strain peak in the slit direction at the same displacement from the center of the blast hole is earlier than that in the vertical slit direction. Comparative analysis of model test 1 and 2, in the direction of the slit, the strain peak generated by the blasting of the strip slotted cartridge is larger than that of the round hole slotted cartridge, and the time to reach the strain peak at the same measuring point also is earlier than that of the round hole slotted cartridge. Based on this analysis, it is preliminarily concluded that the change of the shape of the slit will not make the directional fracture blasting effect of the slit cartridge disappear, but will affect the peak value of the stress and strain of the slit cartridge blasting and the time for each point to reach the peak value of the stress and strain. In the following, the data of the peak stress and strain of each measuring point in the two model tests are filled in Table 1, and the influence of the slit shape on the strain value is further analyzed.

**Table 1:** Measured values of strain of slit charge blasting test with different slit shapes.

Model number		measuring point	peak strain/με	stress peaks/MPa
1	Slitting direction	1	2613.61	94.08
		2	2025.63	72.92
		3	1675.19	60.30
	The direction perpendicular to the slit	4	2454.86	88.37
		5	1821.96	65.59
		6	1437.56	51.75
2	Slitting direction	1	3215.63	115.76
		2	2546.78	91.68
		3	2098.55	75.55
	The direction perpendicular to the slit	4	2441.62	87.89
		5	1775.06	63.90
		6	1388.09	49.97

**Figure 11:**
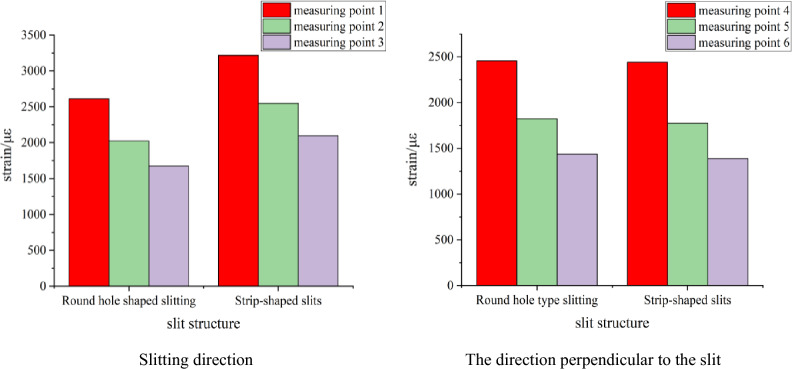
Change of strain peak at each measuring point under different slit shapes.

According to Table 1, the kerf shape-strain peak diagram of each measuring point is drawn, as shown in Fig. 11. The ratio of the strain peak of the first measuring point in the slit direction of the charge blasting under each slit shape to the strain peak of the first measuring point in the vertical slit direction (hereinafter referred to as the ‘strain peak ratio’) is used to measure the size of the directional energy accumulation and loss reduction effect of this slit charge.

It can be seen from Fig. 11 that in the direction of the slit, the strain peak generated by the strip slit charge blasting at each measuring point is greater than that of the round hole slit charge blasting, while the strain peak distribution in the vertical slit direction and the slit direction is just the opposite. According to the analysis of the blasting mechanism and energy transfer theory of the slotted cartridge, the total energy generated by the two kinds of cartridge blasting is the same. Due to the diversion effect of the slit, the blasting energy will be transmitted preferentially from the slit direction. However, because the weak surface created by the strip slit is larger than that of the circular hole slit, the energy diversion effect of the strip slit cartridge blasting in the slit direction is stronger. Because the circular-hole slotted pipe has a small weak surface and a better stability of the pipe body, the binding force on the blasting energy is stronger than that of the strip-shaped slotted pipe, resulting the blasting energy is more consumed in the destruction of the slotted pipe. Based on the above reasons, the strain peak generated by the strip slit charge blasting in the slit direction is larger than that of the round hole slit charge blasting. For the non-slitting direction, according to the law of conservation of energy, the more energy accumulated in the direction of the slit during blasting, the smaller the blasting energy acting on the non-slitting direction. Therefore, the strain value produced by the strip slit charge blasting in the non-slitting direction is smaller than that of the round hole slit blasting. Combined with Table 1, it is calculated that the strain peak ratios of round-hole slit and strip-shaped slit cartridge blasting are 1.065 and 1.317, respectively, and the latter is larger, which is 23.7% higher than the former.

According to the above analysis, it can be seen that the blasting of two kinds of slotted cartridges in the experiment has the effect of directional energy accumulation and damage reduction; but the directional energy accumulation and damage reduction effect of strip slotted cartridge blasting is better.

#### Analysis of blasting crack of slotted cartridge under different slotted shapes

**Figure 12:**
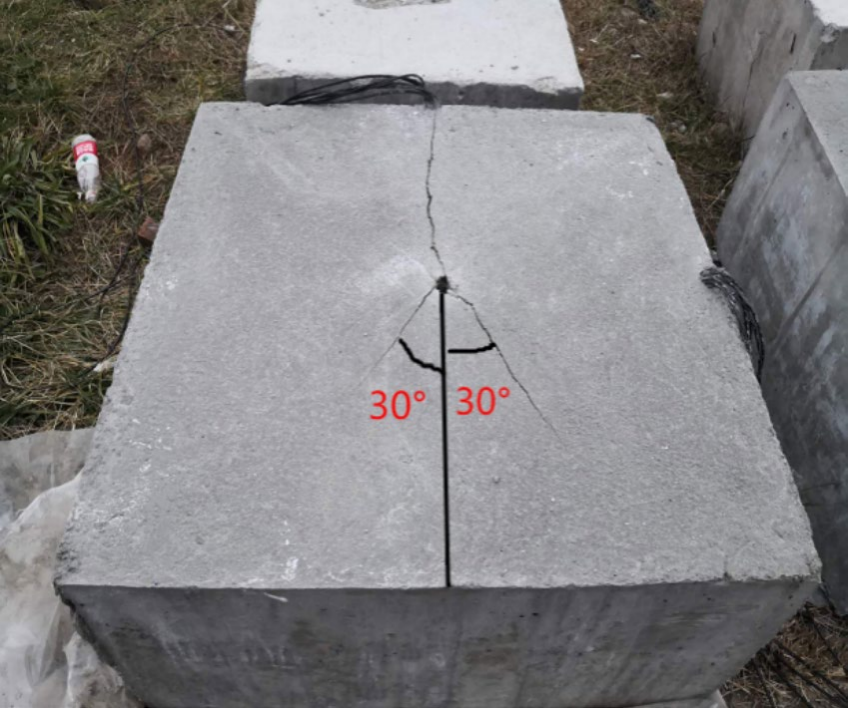
Blast-induced crack distribution in Model 1.

**Figure 13:**
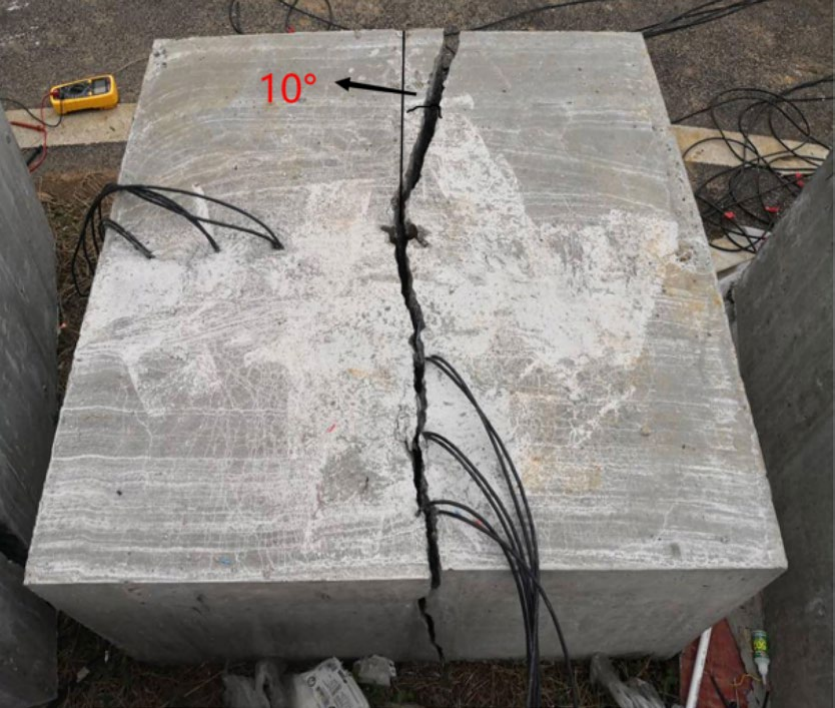
Blast-induced crack distribution in Model 2.

The distribution of blasting cracks in rock mass is the external manifestation of blasting energy transfer, and its expansion direction represents the direction of blasting energy transfer to a certain extent. The propagation length and width can be used to represent the size of the blasting energy. The wider the width of the blasting crack and the longer the propagation length, show the more the blasting energy acting in this direction. In this group of tests, the distribution patterns of blast-induced cracks in the two models are shown in Figs.12 and 13.

As shown in Fig. 12, there are three smaller cracks in the direction of the slit. The main cracks in the upper part of the picture penetrate the boundary of the concrete test block, and the distribution of the two main cracks in the lower part deviates from the established slit direction by about 30 degrees and does not penetrate; no other secondary cracks are produced in the direction of vertical slit. The fracture distribution of the model test block 1 is analyzed. This is because the weak surface of the slit pipe of the circular hole-shaped slit is small, the diversion effect of the slit on the blasting energy is poor, and the blasting stress concentration in the slit direction is not obvious, resulting in poor directional fracture effect of the rock. In addition, the slit pipe of this circular hole type slit has a large compressive capacity, and the blasting energy is excessively consumed when the slit pipe is destroyed, which weakens the blasting energy that promotes crack propagation to a certain extent, resulting in unsatisfactory crack extension.

As shown in Fig. 13, some rock masses around the blast hole collapsed. In the direction of the slit, there is a main crack running through the upper and lower boundaries of the test block, with a width of 1–3 cm, and the extension direction of the upper part of the main crack deviates from the established direction by about 10°. No other secondary cracks were produced in the vertical slit direction, and the directional fracture blasting effect of the model test was good. The crack distribution of model test block 2 is analyzed. When the shock wave generated by the slotted cartridge blasting acts on the blast hole, the surrounding of the blast hole is subjected to strong compressive stress, which is much larger than the dynamic compressive strength of the rock mass, so that part of the rock mass near the blast hole exhibits crushing or caving failure mode. Compared with the circular slit, when the slit shape is a strip slit with a slit angle of 20°, the weak surface of the slit tube is obvious, the slit diversion effect is good, and most of the blasting energy gathers to the rock mass in the slit direction to do work, forming an ideal directional main crack. Because most of the blasting energy is concentrated in the direction of the cutting seam, the blasting energy in the non-cutting direction is reduced, and the cutting pipe weakens the blasting energy in the non-cutting direction, so the damage of the blasting energy to the rock mass in the non-cutting direction is reduced, and the integrity of this part of the rock mass is protected. In addition, according to the theory of rock fracture mechanics, it can be seen from the propagation morphology of rock cracks that the cracks extended under blasting load are mainly type I cracks.

In summary, compared with the round-hole slotted cartridge, the directional energy accumulation and loss reduction effect of the strip slotted cartridge blasting are better, the peak stress and strain generated in the slit direction are larger, the peak stress and strain generated in the non-slit direction are smaller, the distribution of the main cracks in the directional fracture is better, and the secondary cracks are less. According to the test results, it can be concluded that the directional fracture control blasting effect of strip slit cartridge is better.

### Study on the influence of slit angle on blasting effect

**Figure 14:**
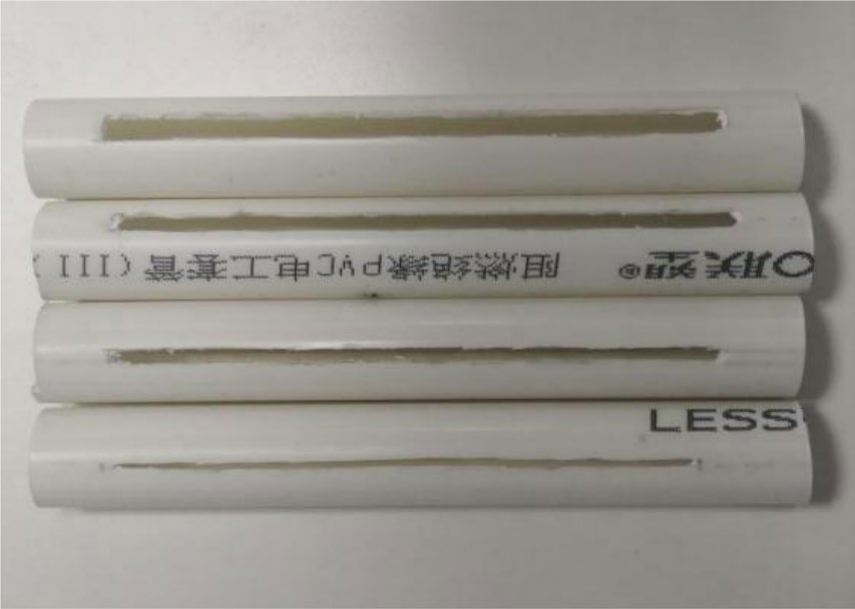
Four slit tubes with different slit angles.

Based on the first set of test results, based on the slit charge with strip slit shape, the experimental study on the influence of slit angle on directional fracture blasting effect was carried out. The slit angle is used as a variable, and the other test parameters and processes are the same as the first set of tests. The test slit pipe is shown in Fig. 14 below. The slit angles are 10°, 20°, 30° and 40° respectively, and the corresponding test block numbers are No. 3, No. 2, No. 4 and No. 5 respectively. The No. 2 model test has been completed in the first set of tests and is not repeated.

#### Analysis of the influence of slit angle on dynamic strain value

**Figure 15:**
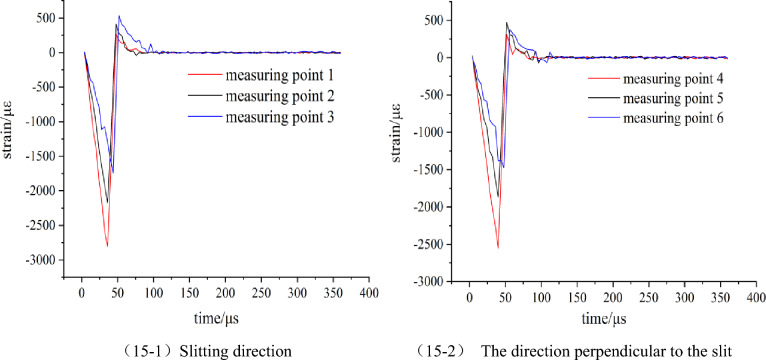
Strain waveform diagram of model 3.

**Figure 16:**
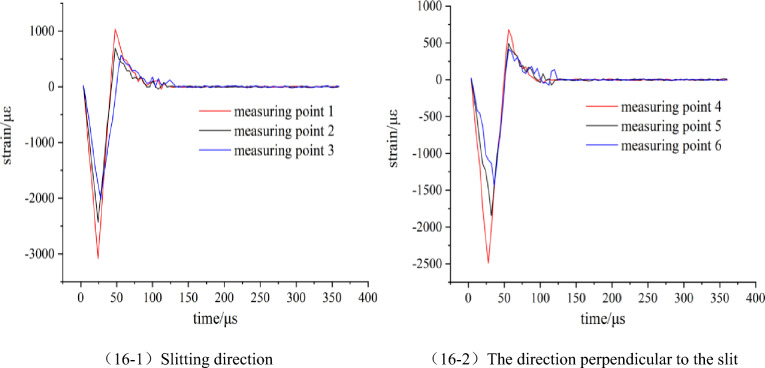
Strain waveform diagram of model 4.

**Figure 17:**
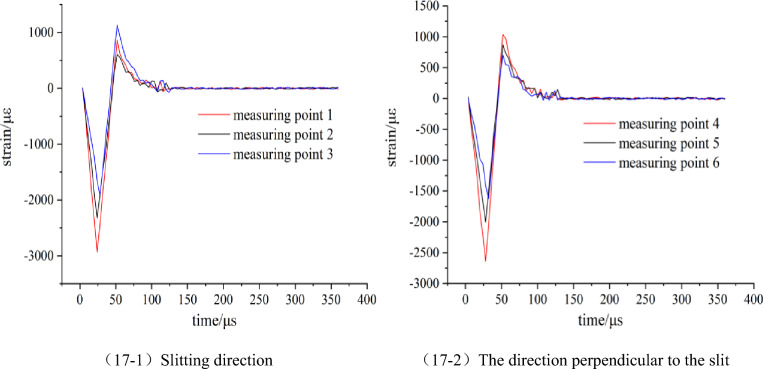
Strain waveform diagram of model 5.

The explosion stress and strain data measured by the second group were processed and plotted to obtain the dynamic strain waveform of each model blasting as shown in Figs. 15, 16 and 17.

By comparing and analyzing Figs. 10 and 15, 16, 17, it is found that the variation law of the strain waveform generated by the blasting of the strip slotted cartridge is similar as a whole. Each measuring point of the rock mass is first subjected to blasting compressive stress and then subjected to blasting tensile stress. The duration of compressive stress is basically within 0–50 μs, while the duration of tensile stress is mostly within 50–150 μs. The duration of tensile stress is longer than that of compressive stress. The existence of the slit tube makes the time to reach the strain peak in the slit direction earlier than that in the vertical slit direction. The time for the dynamic strain to reach the peak in the slit direction gradual shortening with the increase of the slit angle, and the two are negatively correlated. The analysis is due to the wrapping effect of the slit tube in the non-slit direction on the detonation gas and the weakening effect on the shock wave, resulting in the delay of the blasting energy acting on the hole wall rock when the slit angle is small. The difference between the strain waveform of each model is mainly that the peak strain of each measuring point changes with the change of the slit angle, and its size is not the same. The peak strain of each measuring point is extracted from the dynamic strain waveform of each model test, and the Table 2 is shown.

**Table 2:** The measured results of blasting stress and strain peak of each model test block.

Model number		Measuring point	Peak strain (με)	Stress peaks (MPa)
3	Slitting direction	1	2806.23	101.02
		2	2174.22	78.27
		3	1746.07	62.86
	The direction perpendicular to the slit	4	2552.70	91.89
		5	1868.58	67.26
		6	1472.43	53.01
2	Slitting direction	1	3215.63	115.76
		2	2546.78	91.68
		3	2098.55	75.55
	The direction perpendicular to the slit	4	2441.62	87.89
		5	1775.06	63.90
		6	1388.09	49.97
4	Slitting direction	1	3082.69	110.97
		2	2432.49	87.56
		3	2006.46	72.23
	The direction perpendicular to the slit	4	2491.56	89.69
		5	1843.75	66.37
		6	1439.97	51.83
5	Slitting direction	1	2936.82	105.72
		2	2325.96	83.73
		3	1904.72	68.57
	The direction perpendicular to the slit	4	2637.94	94.96
		5	2002.46	72.08
		6	1621.68	58.38

According to the data in Table 2, it is clear that the peak value of stress and strain decreases with the distance from the measuring point to the center of the blast hole. In addition, at the corresponding measuring points, the strain peak in the slit direction is always larger than that in the vertical slit direction.

**Figure 18:**
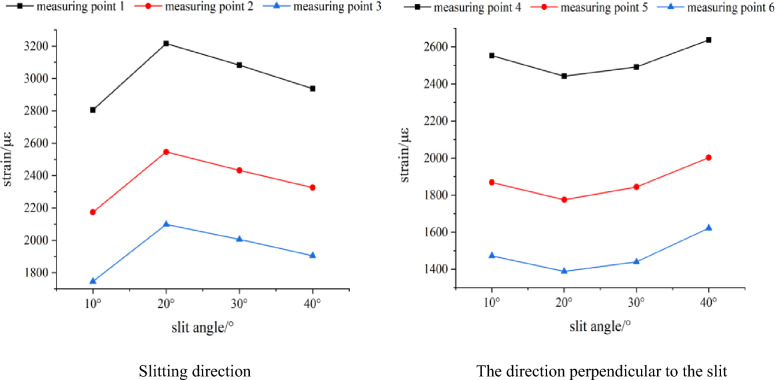
The variation curve of strain peak with slit angle.

Based on Table 2, the slit angle-strain peak diagram of each measuring point is drawn, as shown in Fig. 18.

According to Fig. 18, in the direction of the slit, when the slit angle is 20°, the peak strain of each measuring point is the largest, followed by the slit angle of 30°. On the whole, the peak strain of each measuring point increases first and then decreases with the increase of the slit angle. When the slit angle is in the range of 10°–20°, the strain peak gradually increases with the increase of the slit angle, which is positively correlated, and when the slit angle is in the range of 20°–40°, it is negatively correlated. In the vertical slit direction, the strain peak of each measuring point decreases first and then increases with the increase of the slit angle, which is opposite to the slit direction. The corresponding slit angle is 20° when the strain peak of each measuring point is the minimum value, and the corresponding slit angle is 40° when the strain peak of each measuring point is the maximum value. According to the order of slit angle from small to large, the strain peak ratios of 1 measuring point and 4 measuring points of each slit angle are calculated to be 1.099, 1.317, 1.237 and 1.113 respectively. When the slit angle is 20°, the directional energy accumulation and loss reduction effect of slit cartridge controlled blasting are the best.

The results are analyzed from the perspective of the blasting mechanism of the slotted cartridge and the blasting energy transfer theory. When the slit angle is between 10° and 20°, the notch made by the slit angle of 10° is too small, and the effect of the detonation gas jet is not obvious. The blasting energy reflected by the blasting shock wave after passing through the slotted tube cannot be effectively transmitted and superimposed in the direction of the notch, resulting the blasting shock wave and detonation gas energy transmitted preferentially from the slit direction are small, and the strain peak generated is low. As the slit angle increases to 20°, the energy of blasting gas and blasting shock wave that can be gathered in the slit direction increases significantly, so that the strain peaks of each measuring point in the slit direction increase. When the slit angle is in the range of 20°–40°, with the continuous increase of the slit angle, most of the detonation products are directly emitted from the slit mouth. The blasting shock wave and detonation gas energy radiation surface in the slit direction is wide and strong diffraction occurs at the slit mouth, so that part of the energy acts on the rock mass between the slit direction and the vertical slit direction. The blasting energy acting on the slit direction is weakened to a certain extent, and the strain peak generated also decreases.

#### Analysis of the influence of slit angle on the propagation of blasting cracks

**Figure 19:**
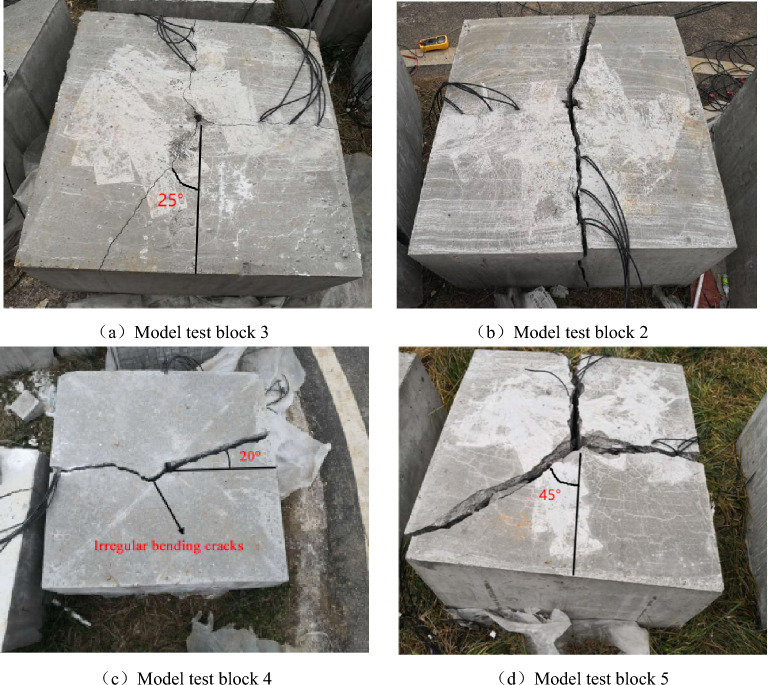
Blast crack distribution of each model test block.

According to the fracture mechanics of rock, it can be seen from Fig. 19 that the fracture type of rock crack caused by slotted cartridge blasting is compound crack propagation mainly with type I crack.

As shown in Fig. 19a, in the upper part of the figure, there are small main cracks extending from the blast hole to the boundary of the test block in the direction of the slit. In the lower half of the diagram, there is a small main crack about 25° to the left of the slit direction. There is a secondary crack smaller than the main crack in the vertical slit direction. The overall directional fracture blasting effect of the model is poor. This phenomenon is analyzed. When the cutting angle is 10° although the blasting energy will be preferentially transmitted from the direction of the cutting seam, due to the small opening of the cutting seam, when the explosion shock wave propagates in this direction, a large part of the shock wave encounters the cutting seam tube, and the explosion shock wave will be reflected and refracted, resulting in a lower intensity of the shock wave acting on the hole wall in the direction of the cutting seam, which in turn causes the initial directional crack of the hole wall to be small and the direction to be offset. In addition, due to the small amount of detonation gas transmitted from this small notch, the effect of detonation gas wedge is not obvious, which leads to the small propagation width of the main crack. Based on the above analysis, it can be seen that the energy accumulation effect in the slit direction is not good, so the blasting energy intensity acting in the vertical slit direction is also large, which makes the vertical slit direction also produce small secondary cracks.

As shown in Fig. 19c, there is a main crack running through the two boundaries in the slit direction of the model test block, with a width of about 1–2 cm. The main crack in the right half of the picture deviates from the slit direction by about 20°, and the main crack in the left half has a small part of the twist near the hole and a fine crack next to it. There are no blast cracks in other places, and the directional fracture control blasting effect is general. The effect is analyzed. When the slit angle is 30°, the energy accumulation effect in the slit direction is good, and the crack is wide and runs through the test block. The main crack offset is considered because the slit port is large, which leads to the weakening of the constraint force of the slit tube on the blasting energy, resulting in a slight expansion of the energy action surface transmitted from the slit port, and the initial directional crack initiation angle is offset, which makes the subsequent crack propagation slightly deviate. For the left part of the figure, there are some irregular main crack. The reasons may be: (1) There are primary defects in the internal structure of the rock mass here, so when the blasting energy is acting in this direction, it is more likely to be destroyed here; (2) It is caused by the compression and tension of blasting stress wave. The author thinks it is more likely to be caused by the first reason.

As shown in Fig. 19d, at the slit direction of the model, the vertical slit direction and the slit direction of the lower half of the figure at 45° to the left, a penetrating crack with similar width appears, and the directional fracture blasting effect of the slit cartridge is poor. The phenomenon is analyzed. When the slit angle is 40°, the proportion of the weak surface of the slit is too large. Although most of the explosion energy is preferentially ejected from the slit, it also causes the radiation range of the shock wave surface to be too wide, the damage area to the hole wall is large, and the initial crack orientation is not accurate. In addition, the too large slit port reduces the stability of the slit tube and the binding force on the detonation gas transfer path. The transmission of blasting energy at the slit port is not concentrated, and some energy is dispersed to the angle between the slit direction and the vertical slit direction. Therefore, the blasting crack distribution phenomenon shown in Fig. 19d appeared in this blasting.

Combined with the dynamic strain value of each measuring point and the crack distribution of the concrete model test block after blasting under different slit angles, it is concluded that in the controlled blasting of the slotted cartridge, under the premise of ensuring that other influencing factors remain unchanged, there is an optimal slit angle in theory, which can make the blasting effect of the slotted cartridge reach the best. In this experiment, when the slit angle is 20°, the directional fracture blasting effect of the slotted cartridge is the best.

## Engineering applications

### Project profile

**Figure 20:**
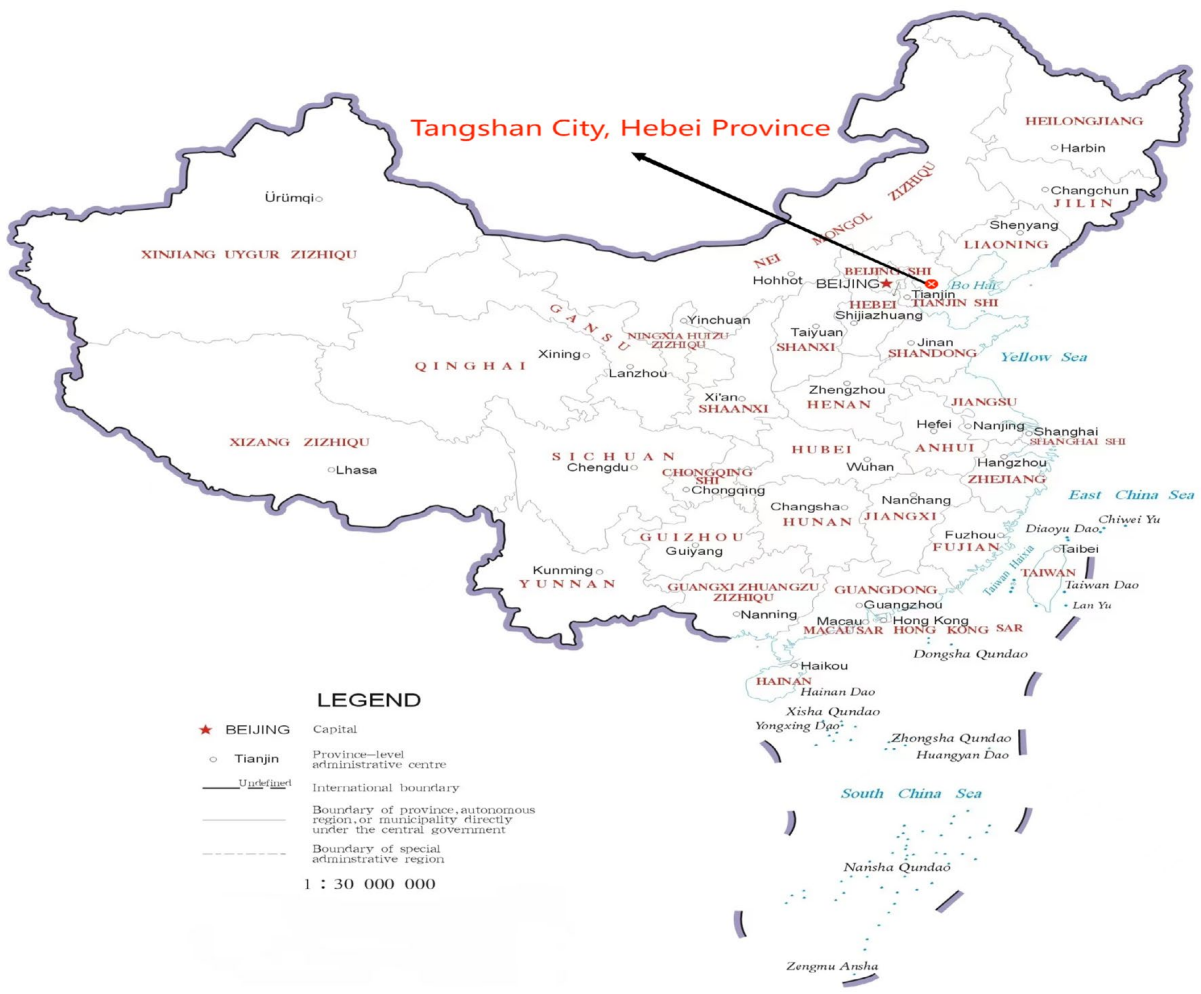
Location of engineering test project.

The blasting test was carried out in the abandoned mining area of Tangshan City, Hebei Province (see Fig. 20). The geological structure in the mining area is simple and no large-scale fault structure is found. The main lithology is gray thick layered chert band dolomite and chert-bearing nodular limestone dolomite. The rock mass structure is mainly thick or medium thick structure, and the rock is hard rock. Because the slope of this blasting is close to the nearby factory road and cement plant, the control requirements for blasting vibration and flying stone are high. In addition, in order to prevent slope collapse and facilitate the later greening engineering treatment of the slope, higher requirements are put forward for the stability and integrity of the slope after blasting. Therefore, it is proposed to use the slotted cartridge pre-splitting blasting technology in the treatment of some slopes to obtain a good directional fracture blasting effect of rock mass, reduce the damage of blasting energy to the protected rock mass, reduce the damage of vibration to distant buildings, and avoid unnecessary safety accidents and disputes.

### Slotted cartridge blasting design and application

**Figure 21:**
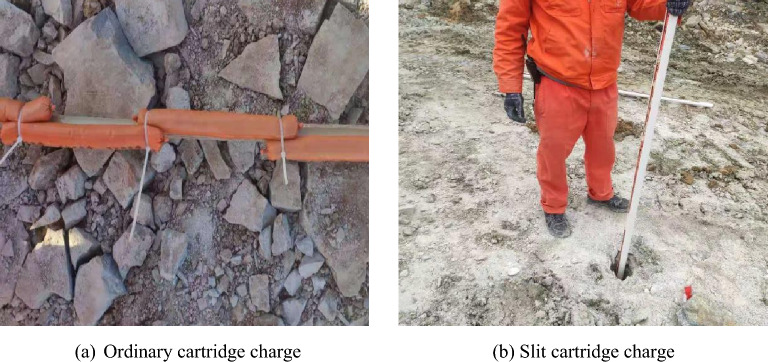
The scene diagram of loading explosives.

In order to test the superiority of the control blasting effect of the best slotted structure slotted cartridge, the blasting effect of the slotted cartridge pre-splitting blasting is compared with the original ordinary pre-splitting blasting. The parameters of the two pre-splitting blasting are designed as Table 3. The inclination angle of the blast hole is consistent with the slope design of 60° and the hole depth is 5 m. The hole length is 6 m. According to the actual construction conditions of the project, a hard PVC pipe with an outer diameter of 40 mm, a wall thickness of 2 mm, and an inner diameter of 36 mm was selected to make a slit pipe, and a No. 2 rock emulsion explosive with a diameter of 32 mm was selected as the explosive material. The above model test shows that when the decoupling coefficient is 1.8, the slit shape is a strip slit, and the slit angle is 20°, the directional fracture blasting effect of the slit cartridge is the best. Combined with the research data^7^, it can be seen that, the slit angle of the slit cartridge blasting should increase with the increase of the decoupling coefficient of the charge. Therefore, based on the model test results and the actual construction situation, the slit tube with slit shape of strip slit and slit angle of 23° was selected to make slit cartridge in this blasting test. The scene of loading explosives is shown in Fig. 21. Due to the influence of blasting materials, construction machinery and other factors, there is a slight difference between the parameters of the slit pipe applied to the construction site and the optimal parameters of the slit pipe obtained by the model test, but this does not affect the verification of the model test results by the field application results.

**Table 3:** Different charge pre-splitting blasting parameters.

Blasting parameters	Blasting plan	
	Presplitting blasting of common cartridge	Presplitting blasting of slotted cartridge
Hole diameter (mm)	90	90
Hole spacing (m)	1	1.3
Charge uncoupling coefficient	2.8	2.8
Linear charge density (kg/m)	0.8	0.8
The way of loading explosives	Gap charging	Gap charging
Detonation mode	Back detonation	Back detonation

### Application result

**Figure 22:**
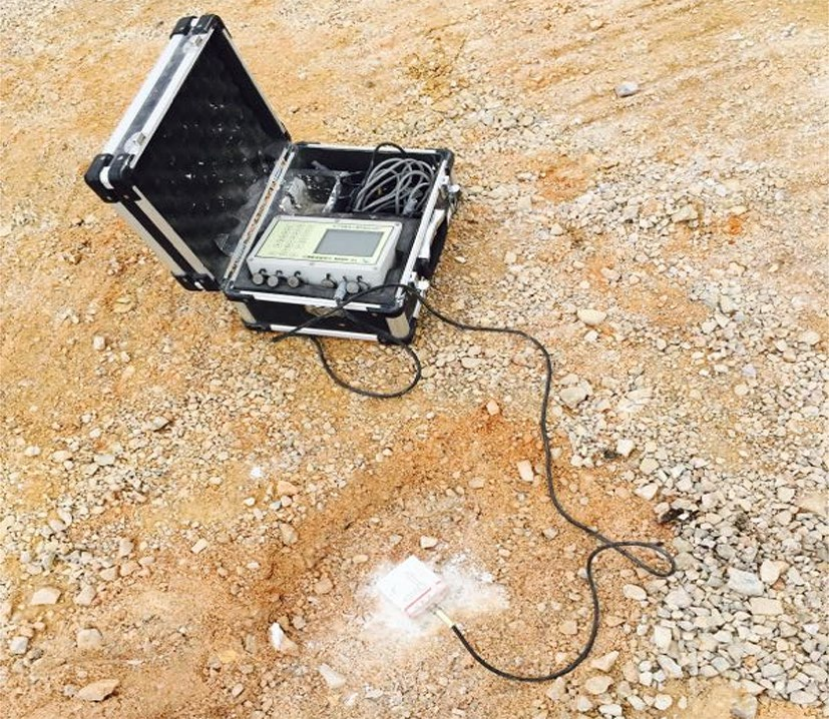
Field vibration measurement diagram.

**Figure 23:**
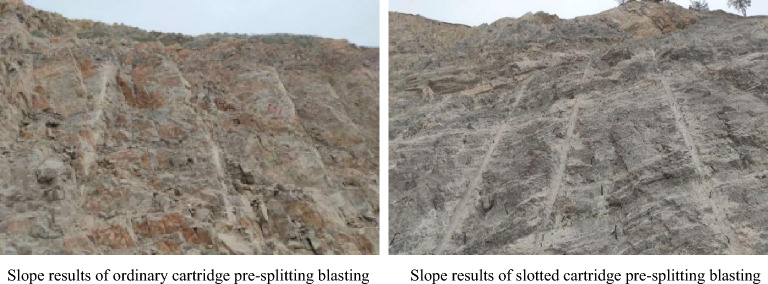
Slope effect diagram after blasting.

In two repeated tests, the blasting vibration intensity is monitored using a TC-4850 blasting vibration meter, as shown in Fig. 22. After blasting, the half hole rate of blasting hole and slope roughness were measured using a tape and ruler, respectively. The measured results of the controlled blasting effect of the different cartridges are listed in Table 4. The slope roughness is shown in Fig. 23.

**Table 4:** Pre-splitting blasting effect of different cartridges.

The serial number of the test	Slope control blasting scheme	Half hole rate of blasting hole (%)	Unevenness of slope (cm)	Blasting vibration velocity peak (cm/s)
First test	Ordinary cartridge	68.2	25.6	0.14
	Slotted cartridge	89.3	11.5	0.05
Second test	Ordinary cartridge	71.6	21.2	0.103
	Slotted cartridge	91.5	13.7	0.046

Based on Table 4 and Fig. 23, it can be seen that the pre-splitting blasting with slotted cartridge is better than that of ordinary cartridge. Compared with the ordinary cartridge pre-splitting blasting, the average roughness of the former slope is 12.6 cm, which is 46.2% lower than that of the latter 23.4 cm. The average half hole rate of the former is 90.4%, which is 20.5% higher than that of the latter (69.9%). The blasting vibration intensity of the former is 0.048 cm/s, which is 60.3% lower than that of the latter 0.121 cm/s.

In summary, the blasting vibration intensity produced by the pre-splitting blasting of the slotted cartridge with the above-mentioned slotted structure is significantly lower than that produced by the pre-splitting blasting of the ordinary cartridge, the half hole rate of the blast hole after the blasting is significantly improved, and the over-under excavation of the protected slope is greatly reduced. It shows that the selection of the slotted structure is reasonable, which verifies the credibility of the model test results. At the same time, it shows that under the condition of reasonable selection of the slotted structure, the use of slotted cartridge pre-splitting blasting is conducive to reducing blasting hazards and improving blasting construction efficiency.

## Conclusion

1. The change of slit shape will not make the energy accumulation and loss reduction effect of slit cartridge disappear, but will affect its effect. The energy-gathering effect in the slit direction and the loss reduction effect in the non-slit direction of the strip slit cartridge blasting are better than those of the round hole slit, which is more conducive to improving the directional fracture blasting effect of the slit cartridge.
2. Under the same conditions of blasting environment, charge amount, charge decoupling coefficient and other blasting factors, with the increase of slit angle, the time for slit charge blasting to reach the peak strain in the slit direction is gradually shortened, and the directional energy accumulation and loss reduction effect of slit charge blasting increase first and then decrease. In the range of 10°–40° selected in this model test, when the slit angle of the slit cartridge is 20°, the strain peak ratio of measuring point 1 to measuring point 4 is 1.317, and the ratio is the largest. The directional fracture blasting effect of slotted cartridge is the best.
3. According to the actual situation of construction, combined with the model test results, the slotted cartridge was applied to the pre-splitting blasting of open-pit slope. After blasting, the half hole rate of blast hole was 90.4%, the slope roughness was 12.6 cm, and the blasting vibration intensity was 0.048 cm/s. Compared with the ordinary cartridge pre-splitting blasting, the half hole rate of the hole is increased by 20.5%, the slope roughness is reduced by 46.2%, and the peak value of blasting vibration is reduced by 60.3%. It can effectively control the slope roughness, reduce the damage of blasting energy to the slope, reduce the construction cost, reduce the blasting hazard and improve the construction efficiency. In addition, the use of slit angle instead of slit width for research is more conducive to the transformation and industrialization of research results.

## Data Availability

All data, models, and code generated or used during the study appear in the submitted article.
